# Influence of Ostwald’s Rule of Stages in the Deracemization of a Compound Using a Racemic Resolving Agent

**DOI:** 10.1021/acs.cgd.1c01426

**Published:** 2022-01-24

**Authors:** Tharit Lerdwiriyanupap, Giuseppe Belletti, Paul Tinnemans, Ruel Cedeno, Hugo Meekes, Elias Vlieg, Adrian E. Flood

**Affiliations:** † Department of Materials Science and Engineering, School of Molecular Science and Engineering, Vidyasirimedhi Institute of Science and Technology, Rayong 21210, Thailand; ‡ Institute for Molecules and Materials, Radboud University, Heyendaalseweg 135, 6525 AJ Nijmegen, The Netherlands; § Department of Chemical and Biomolecular Engineering, School of Energy Science and Engineering, Vidyasirimedhi Institute of Science and Technology, Rayong 21210, Thailand

## Abstract

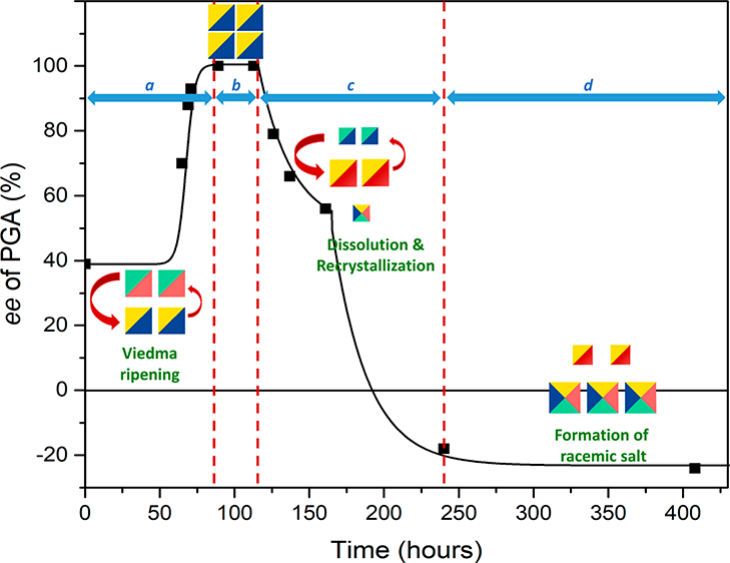

The stereoisomeric system of *rac*-2-phenylglycinamide (PGA) and *rac*-*N*-acetyl tryptophan (NAT) is significant in the application of chiral resolution because it has been shown that this system can be used for enantioseparation of PGA and/or NAT using a novel deracemization route of the conglomerate salt formed. However, it was also found that the conglomerate salt eventually converted into different crystal forms that limited the time available for the separation. Herein, we try to understand the phase conversion occurring in this system using DSC, PXRD, and SC-XRD. The related structures of the salt (two polymorphs of the more stable homochiral (dd- and ll-) salts and one polymorph of the less stable heterochiral (dl- and ld-) monohydrate salts) are demonstrated and discussed relating to their relative stabilities. The successful deracemization was demonstrated using the heterochiral (dl- or ld-) monohydrate salts. However, following Ostwald’s rule of stages, only limited time is available for the deracemization before the metastable compound converts into the more stable homochiral (dd- and ll-) pair. Moreover, the occurrence of the (dd- and ll-) phase always coincides with the formation of yet another phase of the racemic compound containing four components in a crystal. Ostwald’s rule of stages here thus involves three steps and phases and is highly significant during the deracemization of the homochiral species.

## Abstract 2

The evolution of the stereoisomeric system of *rac*-2-phenylglycinamide (PGA) and *rac*-*N*-acetyl tryptophan (NAT) follows a complex route involving three steps in Ostwald’s rule of stages. Deracemization requires termination of the process at the correct time, before more stable crystal forms develop.

## 1 Introduction

Chirality and enantiomerism are common in nature particularly in biological systems. Although enantiomers have identical physical and chemical properties except for optical rotation and some other properties not helpful for enantioseparation in achiral environments, their behavior can be drastically different once inside the human body; i.e., one stereoisomer can be therapeutic, while the other stereoisomer can be inactive or even toxic. Thus, pharmaceutical companies are often required to produce chiral drugs with high enantiopurity to minimize undesirable side effects. Consequently, efficient preparation of enantiopure compounds is of critical importance. Although a vast array of asymmetric syntheses,^1^ biocatalytic syntheses,^2,3^ and advanced separation techniques have been developed such as chiral chromatography^4,5^ and membrane separation,^6^ purification via crystallization still remains the main route in industrial scale manufacturing due to its simplicity and cost-effectiveness. Among the techniques using crystallization, deracemization is an attractive approach since it enables the recovery of a single enantiomeric product from the starting racemic component with yields close to 100%.^7−10^ This can be achieved by simultaneous crystallization–dissolution together with *in situ* racemization in the liquid phase. One process using this mechanism is Viedma ripening.^7,8,11^ However, conglomerate formation is a prerequisite of this process, and only around 10% of chiral compounds satisfy this criterion. Thus, racemates are often converted to conglomerates either via chemical modification^12−14^ or via salt formation^15−17^ before deracemization is performed. Many previous studies have applied the deracemization process for essential pharmaceutical compounds having only one chiral center.^8,12,13,15,17−21^ The application of deracemization has recently been extended to compounds that contain more than one chiral center; however, the number of studies is still limited.^22,23^

**Figure 1:**
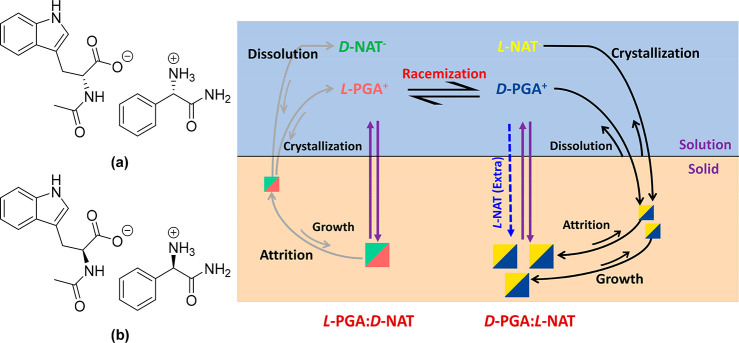
Left: Chemical structures of the two enantiomers of the salt; d-PGA/l-NAT (a) and l-PGA/d-NAT (b). Right: Scheme demonstrating the conversion of this salt into a single stereoisomer.^23^ Viedma ripening deracemization has previously been implemented to obtain enantiopure PGA using the racemic conglomerate salt d-PGA/l-NAT monohydrate (and its mirror counterpart) formed from *rac*-PGA and *rac*-NAT.

In our previous work,^23^ we have demonstrated a chiral purification approach for a racemic compound via salt formation with a racemic resolving agent. A successful example was shown using a racemic conglomerate salt of *rac*-2-phenylglycinamide (PGA) and *rac*-*N*-acetyl tryptophan (NAT). By implementing Viedma ripening, the solid phase species can be completely converted into a single stereoisomer. This approach was used in the enantiopurification of PGA which is an important drug precursor^24^ and a useful chiral auxiliary in organic synthesis.^25−27^ The chiral purification was successfully achieved using racemization of only the chiral center of *rac*-PGA. Since NAT is not racemizable under these conditions, there will not be any conversion of the NAT to the form required to create the desired salt form. Thus, in order for the process to be possible, an excess amount of *rac*-NAT is necessarily required (Figure 1). This process resulted in an excellent enantiopurity of nearly 100%.

We have observed, however, that when the deracemization process is allowed to continue for longer periods, the enantiomeric excess (*ee*) gradually drops from the value of 100%. Analysis of the resulting crystals reveals that this is caused by phase transformations to more stable ones, following Ostwald’s rule of stages.^28^ This rule states that, during the crystallization, the less stable (or kinetically favored) form usually crystallizes first and then is spontaneously converted to the more stable form by recrystallization. In this work, we investigated the phase transformations in the PGA/NAT system, leading to the discovery of two new polymorphic forms and one solvate. Using X-ray crystallography, we then elucidated their crystal structures which are hitherto unknown. This work highlights that in designing deracemization processes, assessment of possible phase transformations is crucial as it could pose a serious threat to the deracemization efficiency.

## 2 Experimental Section

*rac*-PGA and l-PGA with 95% purity were purchased from ABCR GmbH & KG. d-PGA with 98% purity was purchased from AKSci. *rac*-NAT with 98% purity and l-NAT with 99% purity were purchased from Sigma-Aldrich. d-NAT with 98% purity was purchased from TCI. Salicylaldehyde with 99% purity was purchased from Fisher Scientific. All solvents were acquired from VWR International. All chemicals were used as received. The salt preparation was implemented following the procedure reported in the literature^23^ with quantitative yield. The formation of the racemic conglomerate salt was further confirmed by the agreement of the experimental powder X-ray diffraction (PXRD) pattern and the simulated pattern from the reported structure (CCDC 2093020) (see Figure S1, Supporting Information).

The racemic conglomerate salt mixture was prepared from *rac*-PGA and *rac*-NAT. Single crystals of the stereomerically pure salts were prepared from d-PGA and l-NAT (dl-salt) and from l-PGA and d-NAT for its mirror-image pair (ld-salt). On the contrary, the salt from d-PGA and d-NAT (dd-salt) and its mirror-image pair (the l-PGA and l-NAT salt or ll-salt) were first obtained as an oil phase when using MeOH as a solvent despite the absence of the solvent in the salt product as confirmed by ^1^H NMR. The properties of these starting compounds were investigated using PXRD, SC-XRD, and DSC.

Viedma ripening experiments were performed starting from seeds of the metastable conglomerate dl-monohydrate salt. 2.25 g (5.43 mmol) of the racemic salt mixture, 50% excess of *rac*-NAT (0.67 g, 2.72 mmol), seed crystals of dl-monohydrate salt (0.12 g, 0.29 mmol), 2 g of Ø ca. 2 mm glass beads and an oval PTFE-coated magnetic stirring bar (L 20 mm, Ø10 mm) were added in the presence of 159 μL of salicylaldehyde as a racemization catalyst for PGA in 4 mL of ethanol at 65 °C. The suspension was stirred at 700 rpm, and the solid samples were taken over time to investigate the relationship between the *ee* of both PGA and NAT species and the phase composition during the experiment.

Analysis of the solid samples was performed using PXRD with a Bruker D8 Advance diffractometer. Cu Kα was used as the radiation source (λ = 1.5418 Å). The analysis was performed at 2θ values ranging from 5° to 40° with a step size 0.01° and 0.3 s per step. The phases were further investigated using DSC measurements with a Mettler Toledo DSC1 calorimeter and a high sensitivity sensor (HSS8) combined with LN2 liquid nitrogen cooling, a sample robot, and STAR^e^ software. A few milligrams of sample were sealed in an aluminum pan, and the heat flow was measured as a function of temperature compared to an empty reference pan. The samples were heated in the temperature range of 25–200 °C with a rate of 2 °C/min. The melting temperature was determined as the onset temperature.

The single-crystal structural determination was performed on a Bruker D8 Quest diffractometer with a Mo Kα monochromator (λ = 0.71073 Å). The structures were solved using SHELXT^29^ via direct methods. All reflections were refined with SHELXL-2014 using the least-squares method.^30^ Non-hydrogen atoms were refined with anisotropic displacement parameters, and hydrogen atoms were refined with a riding model.

Suitable single crystals of the most stable phase were selected to investigate the components appearing in the single crystal. A HPLC (Agilent Technologies 1100 series) equipped with a UV–visible diode array detector was used to measure the enantiomeric composition of the NAT species of each crystal using a Lux 5 μm Amylose-1 column with a column of 4.6 mm diameter × 250 mm length. The experiments were performed with a mobile phase containing hexane/2-propanol/trifluoroacetic acid with the ratios of 80%, 20%, and 0.1%, respectively, and with a flow rate of 0.50 mL/min at 35 °C.

## 3 Results and Discussion

**Figure 2:**
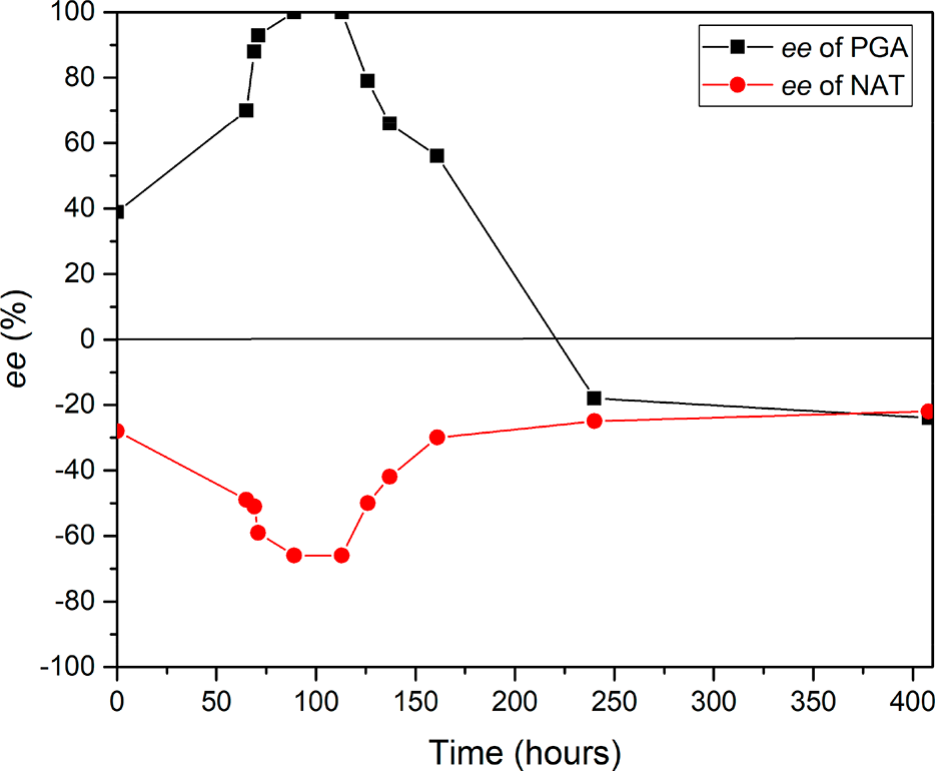
Evolution of *ee* of PGA and NAT in the solid phase during the Viedma ripening experiment when using 50% excess of *rac*-NAT. Note: the dl-salt was used as seeds, and the direction of d-enantiomer was assigned as the direction of positive *ee*.

**Figure 3:**
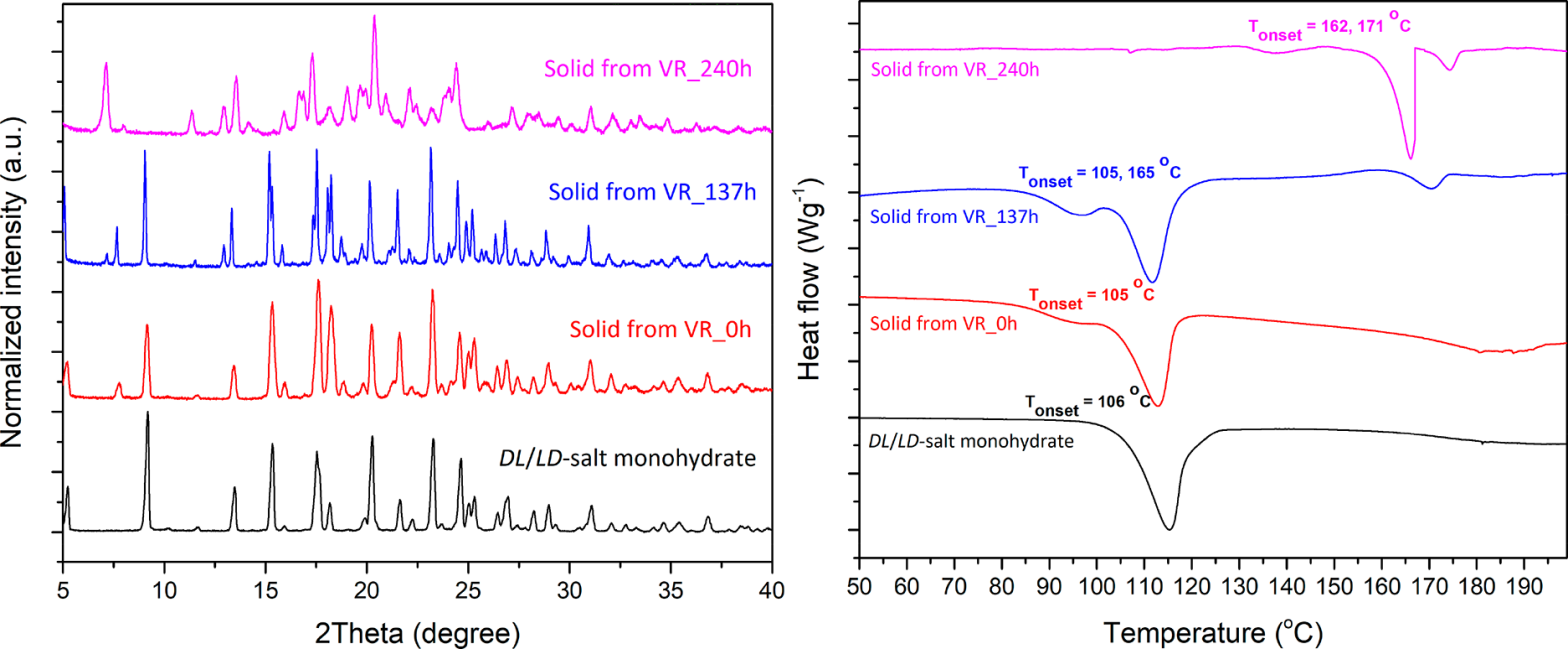
PXRD patterns (left) and DSC thermograms (right) of solid phase obtained from Viedma ripening experiment at 0 h (red), 137 h (blue), and 240 h (pink) compared to the metastable phase of heterochiral (dl- or ld-) monohydrate salt (black).

The evolution of enantiopurity of PGA and NAT species in the solid phase for the simultaneous deracemization of *rac*-salt (containing *rac*-PGA and *rac*-NAT) via Viedma ripening was investigated by HPLC following the method used previously^23^ with details described in Supporting Information. The result is shown in Figure 2. Note that initially the system consisted of a suspension of heterochiral monohydrate salts, i.e., dl- and ld-salts (being equivalent to *RS*- and *SR*-salts, respectively, when considering the systematic use of the CIP system), which crystallize as a conglomerate. The result shows that the solid phase evolves toward the stereopure state, a dl-salt, within ∼90 h leading to an enantiopure PGA species after hydrolysis of the salt. Surprisingly, after about 110 h, the *ee* of PGA starts to decrease from 100% down to ca. −25%. This poses a serious risk to the deracemization efficiency because there is only a limited time window to reach and maintain 100% *ee*. To understand why this happens, we investigated the suspended solids via PXRD and DSC for several time intervals (0 h, 137 h, and 240 h), and the results are shown in Figure 3.

**Figure 4:**
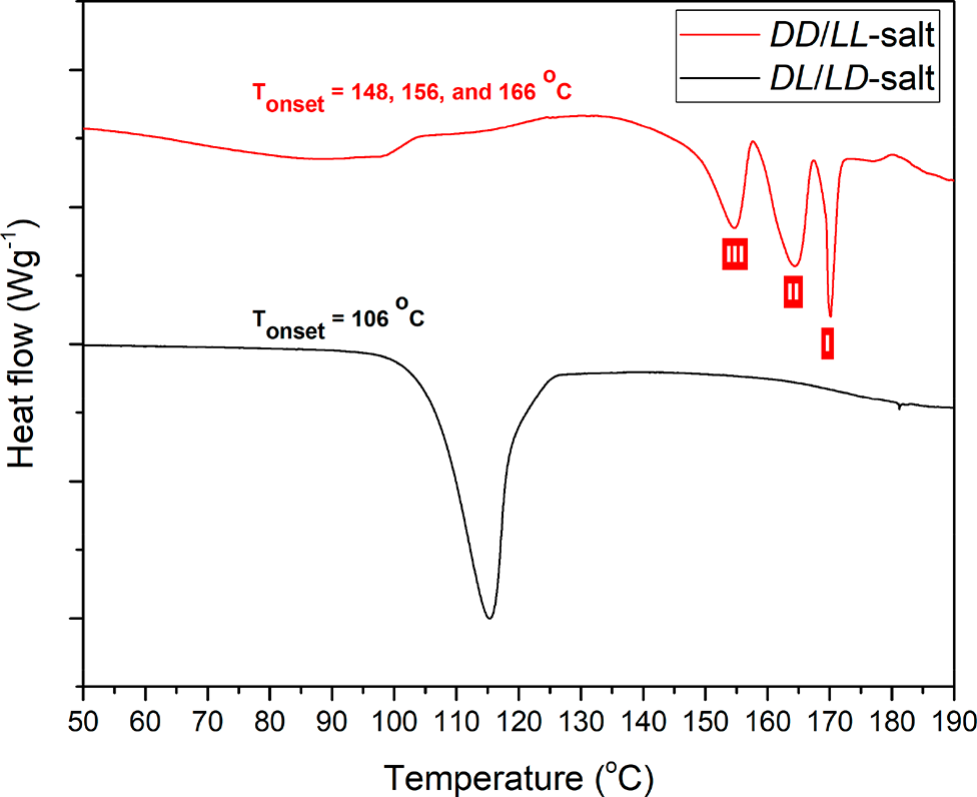
DSC thermograms of the two stereoisomeric pairs, the heterochiral (dl- or ld-) monohydrate salt shown in black and the homochiral (dd- or ll-) salt shown in red. The number in the red square labels the polymorphic forms observed in the homochiral (dd- or ll-) salt.

From the PXRD pattern, some peaks disappear (notably at 9.2°, 15.4°), while some peaks appear (at 7.0°, 12.9°), which is characteristic of phase transformations. This is also supported by the DSC curves which show shifting of the melting temperature and appearance of new peaks. The DSC trace taken at 240 h shows a strong endothermic peak at 162 °C and a further small endothermic peak at 171 °C, indicating that transformations occur between several forms. While the crystal structure of the heterochiral (ld- or dl-) monohydrate salt conglomerate system has already been reported in our previous work,^23^ the structures of the homochiral salt forms (dd- or ll- which are equivalent to *RR*- or *SS*-, respectively, when considering the systematic use of the CIP system) are hitherto unknown. In the preparation of the homochiral stereoisomers in methanol, an oil was first obtained, which then gradually solidified at room temperature after a month. Interestingly, the resulting solid exhibits three melting peaks (Figure 4), which implies that either it starts off as a pure polymorphic form and then undergoes phase transformations to more stable polymorphic forms, or it is a mixture of at least three polymorphic forms. For brevity, we will denote the polymorphs as form I, form II, form III, corresponding to the phase with the highest, intermediate, and lowest melting point, respectively.

**Figure 5:**
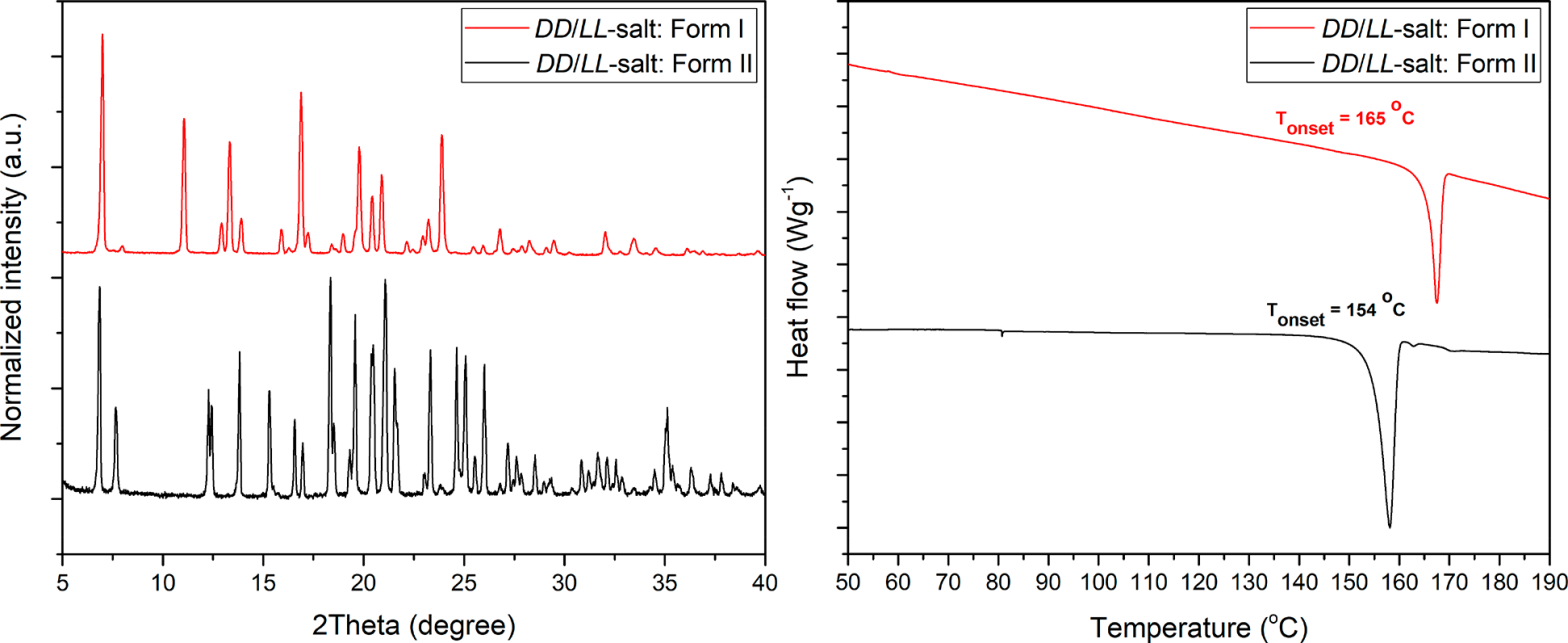
PXRD patterns (left) and DSC thermograms (right) of the two polymorphic forms, I (red) and II (black), of the homochiral (dd- or ll-) salt.

To isolate each polymorph of the homochiral (dd- or ll-) salt, we screened several conditions so that only pure phases of each polymorph would crystallize. For form III, we found that it is very challenging to find the suitable condition where it forms without the concomitant formation of other forms. Indeed, we have attempted various solvents, e.g., MeOH, EtOH, IPA, water, as well as mixed solvents system (chloroform + MeOH and DCM + MeOH), but they were not successful. We were successful in exclusively crystallizing forms I and II. Form II could be obtained after leaving the solid phase (containing three forms, with still some remaining oil) for a month at room temperature, resulting in the pure solid form. Form I could be obtained from fast evaporation in ethanol at room temperature. Each of the pure polymorphic forms I and II was further characterized by PXRD and DSC (Figure 5). The PXRD of these two polymorphic forms are clearly not identical, which confirms the formation of different phases. Further investigation by DSC reveals that the melting temperatures of form I and form II are 165 and 154 °C respectively.

**Figure 6:**
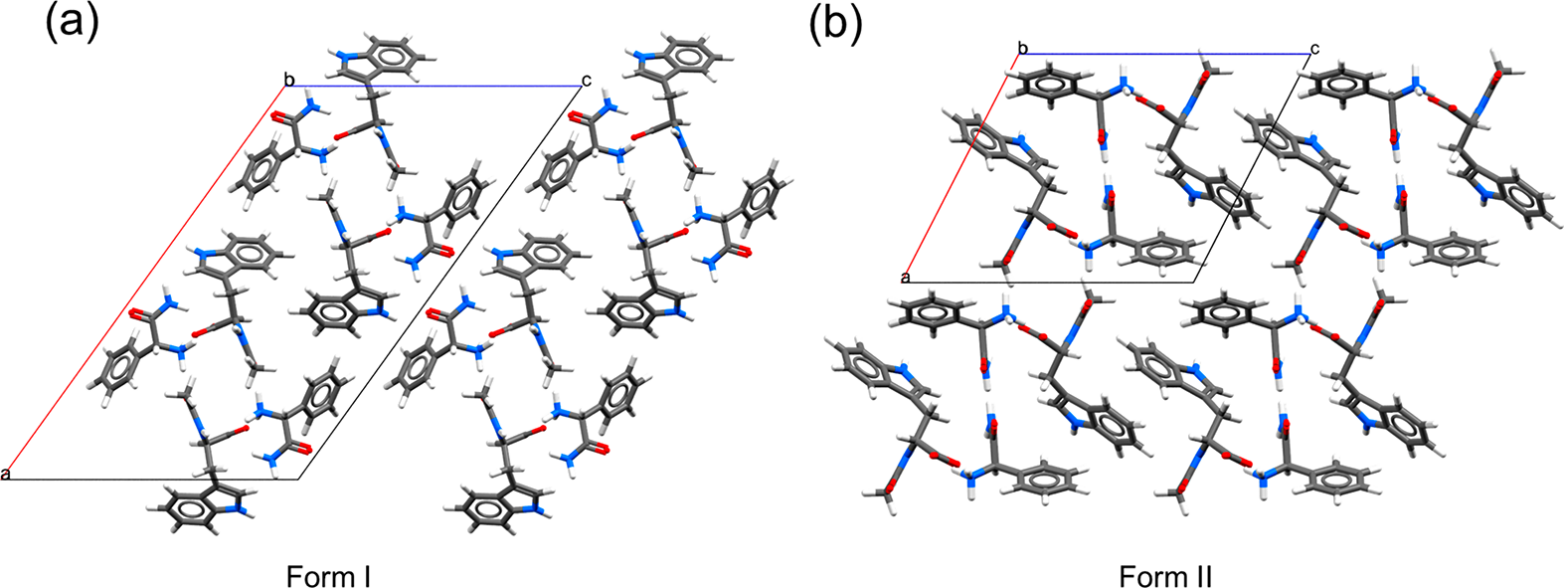
Crystal packing of (a) Form I and (b) Form II of the homochiral (dd- or ll-) salt viewed along the *b*-axis.

To elucidate the crystal structures of form I and form II, we employed single-crystal X-ray crystallography. The pertinent crystallographic information is listed in Table 1, and the crystal packings are illustrated in Figure 6.

**Table 1:** Crystallographic Information for Form I and Form II of the Homochiral (dd- or ll-) Salt

polymorph	Form I	Form II
crystal system	monoclinic	monoclinic
space group	*C*2	*P*2_1_
*a*/Å	26.8930(12)	12.8137(10)
*b*/Å	5.6338(2)	5.8635(5)
*c*/Å	15.9848(7)	14.2626(12)
α/°	90	90
β/°	123.9220(15)	115.201(3)
γ/°	90	90
cell volume/Å^3^	2009.65(15)	969.60(14)
no. of formula units per cell (*Z*)	4	2
density/g·cm^–3^	1.314	1.358
*R*_1_ (*I >* 2σ (*I*))	0.0433	0.0338
*wR*_2_ (all data)	0.1147	0.0938

While both forms are monoclinic, form I and form II crystallize in *C*2 and *P*2_1_ space groups, respectively. Interestingly, although form I has a higher melting point than form II, form II has a higher density (closer packing) than form I. Given that form II was obtained via gradual polymorphic transition while form I was obtained via fast cooling, it appears that the system is enantiotropic. It is also worth noting that the heterochiral (dl- and ld-) monohydrate salts were always observed from crystallization of the salt from solutions containing the four components (*rac*-PGA and *rac*-NAT), even though the newly identified diastereomeric ll- and dd-pair has a melting temperature at least 42 °C higher than these conglomerate monohydrates. The preferential formation of a metastable form is of course an example of Ostwald’s rule of stages. This metastable heterochiral (dl- and ld-) monohydrate salt form is stable enough to allow deracemization to an *ee* of 100%, similar to the results of Spix et al.^31^ However, at some point, conversion to the more stable homochiral (dd- and ll-) salts starts.

Having characterized the four phases involved, we can now analyze their interplay in the Viedma ripening experiment. Reinspection of the PXRD patterns in Figure 3 during the time intervals of the Viedma ripening experiment together with the PXRD patterns in Figure 5 of the two polymorphic forms of the homochiral (dd- or ll-) salt shows that form I of the homochiral salt is the first to appear as evidenced by the good agreement between these two diffraction patterns as well as the melting temperature between these two species (a comparison is shown in Figure S2, Supporting Information). This enantiomeric pair is a conglomerate, just like the starting heterochiral (dl- and ld-) pair, and in principle Viedma ripening should be possible for the new combination as well. Experimentally, however, we see a big drop in the magnitude of the *ee* for both species (Figure 2) as soon as the formation of the homochiral (dd- and ll-) salts start. The likely reason for this is that during the conversion, the dl-salt (which was the dominant solid species from the initial Viedma ripening process) dissolves when the homochiral (dd- and ll-) salts are formed, and during this dissolution the d-PGA will be racemized. Because of the high solubility of the heterochiral (dl- or ld-) monohydrate salt (∼700 mg/mL, see Figure S3, Supporting Information), the solution stays nearly racemic, and the homochiral (dd- and ll-) salts will form in roughly equal amounts. This rapidly decreases the *ee* of the crystals. In fact, since there is an excess of the l-NAT enantiomer, more ll-salt is expected to form, and this leads to the observed reversal in the *ee* of the PGA species. All this occurs during the time interval of 113–240 h of the experiment (Figure 8a, stage c).

After 240 h of the Viedma ripening experiment (Figure 8a, end of the stage c), the metastable conglomerate of the dl-salt had completely disappeared, and the species predominantly existing in the solid phase is the mixture of the two homochiral (dd- and ll-) salts as confirmed by DSC. Moreover, the HPLC result indicates that the direction of the deracemization is biased toward the ll-salt in all of three experiments we performed (see Table S2, Supporting Information) because of the dl-seeds used.

**Figure 7:**
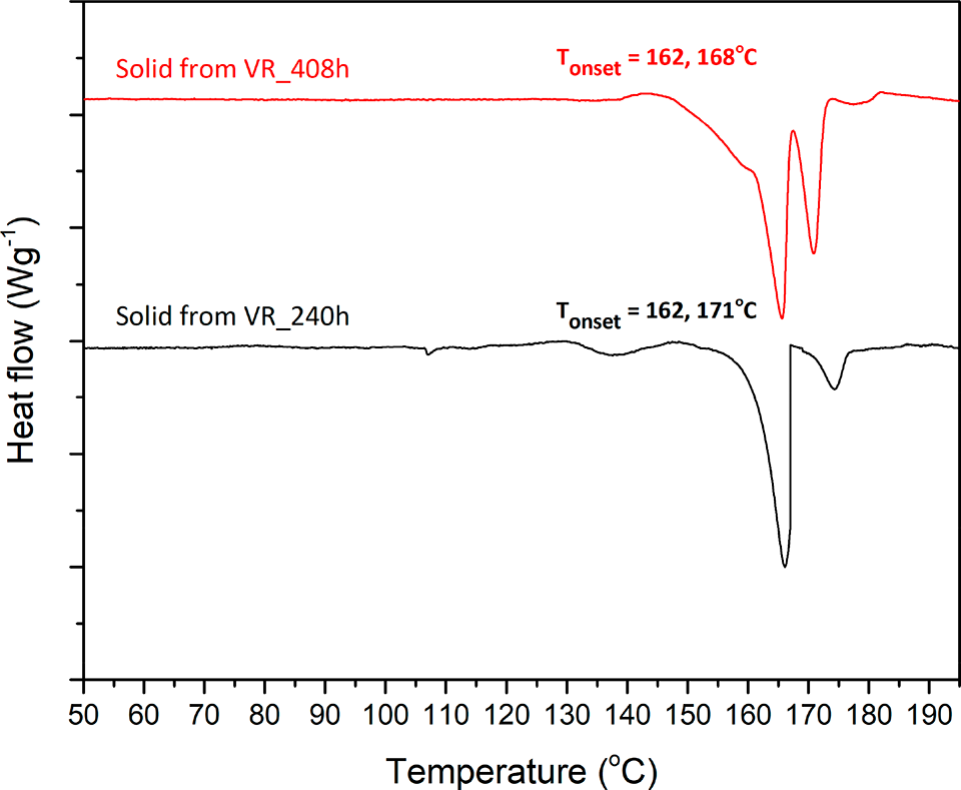
DSC thermograms of the solid product obtained from the Viedma ripening experiment after 240 h (black) and 408 h (red).

**Figure 8:**
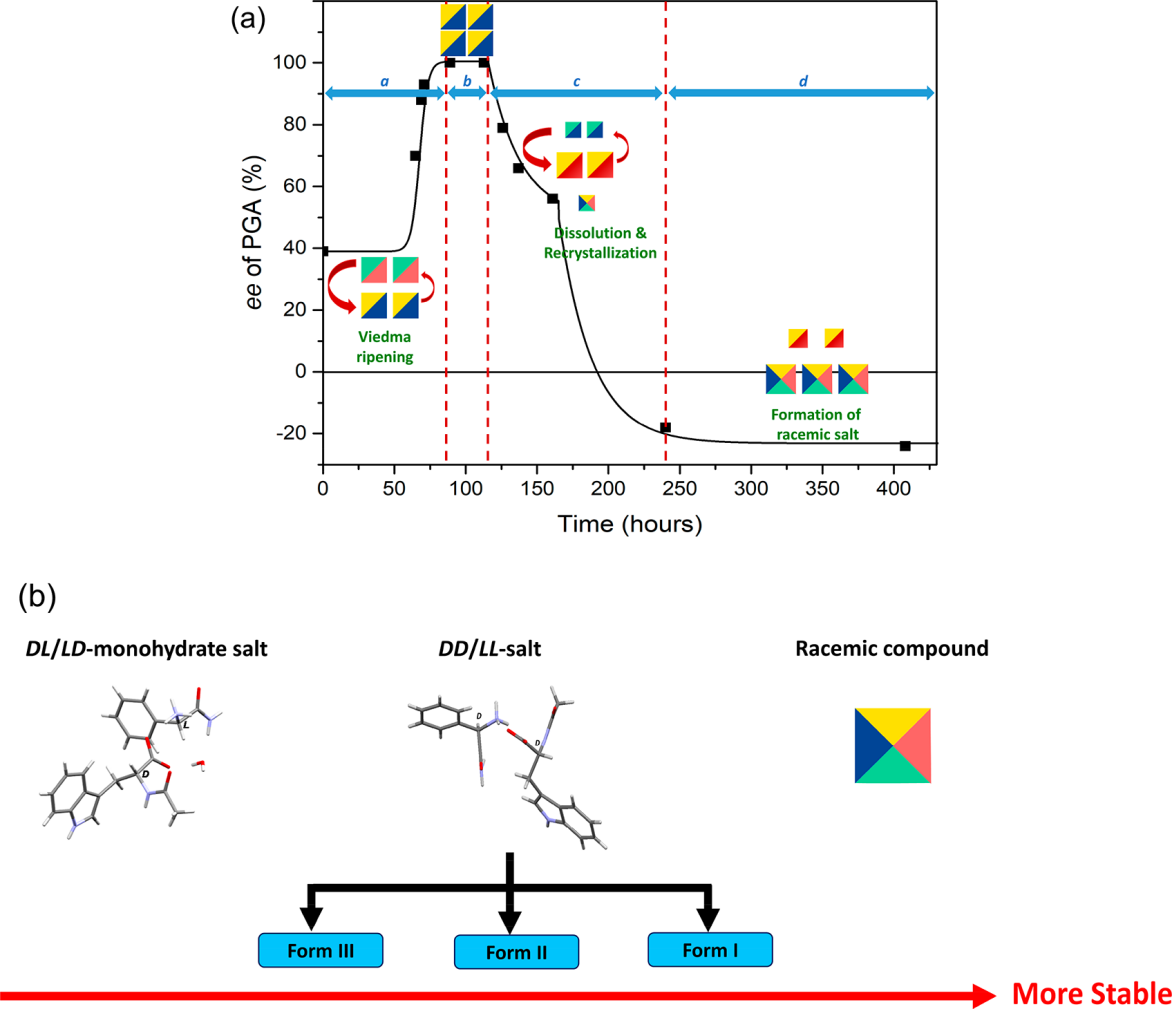
(a) Scheme demonstrating the existing salt species during the time intervals of the Viedma ripening experiment divided into four stages: *a*. evolution of the mixture of heterochiral (dl- and ld-) monohydrate salts to the stereopure dl-salt as induced by the seed crystals, *b*. stereopure state of the dl-salt, *c*. dissolution of the dl-salt and recrystallization of the more stable homochiral (dd- and ll-) salts, *d*. formation of the racemic compound containing d-PGA, l-PGA, d-NAT, and l-NAT in one crystal, (b) comparison of the relative stabilities under the Viedma ripening experiment of the overall species observed in the system of the salt containing *rac*-PGA and *rac*-NAT.

The Viedma ripening experiment of this system was continued until 408 h (Figure 8a, stage d). Since homochiral (dd- and ll-) salts form a conglomerate system, an increase in *ee* is expected to take place, but this does not occur. The higher stabilities of the species present, and therefore their lower solubility, in combination with the excess of *rac*-NAT may make Viedma ripening slower compared to the successful deracemization using the heterochiral (dl- and ld-) monohydrate salts. Indeed, if the kinetics are very slow, an intermediate phase should consist of all material in the form of the ll- and dd-salts, and then with a small excess of ll- salt (since l-NAT is present with some excess). Nevertheless, the dd- and ll-combination fulfills all requirements for Viedma ripening, and, even when it would be slow, eventually deracemization should occur. The reason why this does not happen for this system is that there is yet another phase that forms in this system. This phase, with a melting point around 168–171 °C, does not match with any known phases of these two stereoisomeric pairs. Moreover, it is more pronounced when the experiment proceeded until 408 h as confirmed by DSC (Figure 7). Apart from DSC, the PXRD pattern of the solid product at 408 h of the experiment also displays additional peaks which correspond to a new phase (see Figure S4, Supporting Information). As detailed below, this is a racemic compound with dldl-composition that is more stable than the homochiral (ll- and dd-) compounds. We thus encounter Ostwald’s rule of stages again: the ll- and dd-combination is only a step toward the (presumably) final racemic compound. With a racemic compound as the end product, no deracemization is possible, and thus the *ee* of PGA species stays at a slightly negative value.

To prove that the most stable phase is a racemic compound, we put much effort to investigate the structure, but, unfortunately, it was not possible to obtain a suitable crystal for the X-ray crystallography due to the insufficient thickness of crystals for the diffraction measurement. Therefore, a workaround solution was used to investigate the components of this crystal phase. We selected three different suitable single crystals obtained from the slow regrowth of this solid phase in the saturated solution (see Figure S5, Supporting Information) and determined the enantiomeric composition of the NAT species of each crystal independently by an HPLC technique (see Figure S6 and Table S3, Supporting Information). Although the condition of HPLC cannot determine the enantiomeric purity of PGA, the results show that the enantiomeric excess of the NAT species is 0% (within the reproducibility of the measurement) for all three selected crystals. This indicates that the species contained in the most stable phase is the racemic compound comprised of d-PGA, l-PGA, d-NAT, and l-NAT in one crystal.

To the best of our knowledge, this case is the first example that phase transformations between two diastereomers were observed for the deracemization of the compound containing multiple stereocenters. Although we have seen examples of chiral molecules with one stereocenter exhibiting the metastable conglomerate behavior such as 2′-benzyloxy-1-1′-binaphtalene-2-ol^14,32^ and glutamic acid,^31^ their mechanisms are not directly applicable to the molecules having more than one stereogenic center since the stability of the nonmirror image stereoisomers is always different and needs to be considered. If the deracemization is started with the stereoisomeric pair that is the least stable (i.e., the heterochiral (dl- and ld-) monohydrate salts compared to the homochiral (dd- and ll-) salts for our system), the system can be driven toward the direction of the more stable one. In addition, in our case, the racemic compound is the most stable species (Figure 8b), making the deracemization more challenging since, according to Ostwald’s rule of stages, phase transformations are highly likely to occur during the experiment which takes place over several days. Therefore, in order to avoid problems during the deracemization of this system, some of the following procedures need to be implemented, (i) seeds with a large amount of the desired species because the time to complete the deracemization is crucial for a metastable species, (ii) the synthesis route for the *rac*-salt can be modified since the solvent, temperature, and solvent evaporation approach may affect the lifetime of the metastable state during the deracemization experiment, (iii) the parameters of the deracemization experiment, e.g., the temperature used, the amount of glass beads, the amount of the racemizing agent, and the suspension density should be carefully optimized.

## 4 Conclusion

We have demonstrated that even though deracemization is possible for the salt of *rac*-PGA and *rac*-NAT toward the direction of the heterochiral (dl- or ld-) monohydrate salt, detailed investigations of the relative stability show that the system may not be best suited when considering the practical aspect of the deracemization. Even though the crystallization from an equimolar amount of *rac*-PGA and *rac*-NAT leads to the formation of heterochiral (dl- and ld-) monohydrate salts, owing to their lower stability compared to other species existing in the system (the homochiral (dd- and ll-) salts and the racemic compound) under the same condition, we find that the system eventually evolves toward these more stable phases, following Ostwald’s rule of stages, which could threaten the deracemization efficiency. A decrease in the magnitude of the *ee* for both species after the system had reached the stereopure state of the dl- or ld-salt was registered to be the formation of the more stable homochiral (dd- and ll-) salts which show a much higher melting temperature. The system is still a conglomerate during this conversion between the two diastereomers; nevertheless, the deracemization of homochiral (dd- and ll-) salts is very challenging since Ostwald’s rule of stages still plays a role, making the mixture of dd- and ll- only an intermediate step toward the racemic compound which exhibits the highest stability in this system. Therefore, there is probably not sufficient time to deracemize these species before the racemic compound appears. As the current stereoisomeric system shows, Viedma ripening may be possible using a metastable conglomerate, but this requires a careful investigation of all the crystal forms that can occur and their relative stabilities. Since systems with multiple stereocenters have several potential combinations, such an investigation may also reveal the most suitable form for a deracemization process.

## Supporting Information Available

HPLC analyses; PXRD results; bonding information; solubility data; *ee* determination of NAT in the final compound (PDF)

## Accession Codes

CCDC 2123820–2123821 contain the supplementary crystallographic data for this paper. These data can be obtained free of charge via www.ccdc.cam.ac.uk/data_request/cif, or by emailing data_request@ccdc.cam.ac.uk, or by contacting The Cambridge Crystallographic Data Centre, 12 Union Road, Cambridge CB2 1EZ, UK; fax: +44 1223 336033.
