# Supplementary material for: Influence of Ostwald’s Rule of Stages in the Deracemization of a Compound Using a Racemic Resolving Agent
Source: Cryst Growth Des. 2022 Jan 24;22(2):1459–66. doi: 10.1021/acs.cgd.1c01426 (PMC8815034; doi:10.1021/acs.cgd.1c01426)
Supplement: Supplementary file 1 — cg1c01426_si_001.pdf [file cg1c01426_si_001.pdf]

Supplementary Information

The influence of Ostwald's rule of stages in the deracemization of a compound using a racemic resolving agent

Tharit Lerdwiriyanupap,[1,2] Giuseppe Belletti,[2] Paul Tinnemans,[2] Ruel Cedeno,[3] Hugo Meekes,[2] Elias Vlieg[2*] and Adrian E. Flood[3*]

[1]*Department of Materials Science and Engineering, School of Molecular Science and Engineering, Vidyasirimedhi Institute of Science and Technology, Rayong 21210, Thailand*

[2]*Radboud University, Institute for Molecules and Materials, Heyendaalseweg 135, 6525 AJ, Nijmegen, The Netherlands*

[3]*Department of Chemical and Biomolecular Engineering, School of Energy Science and Engineering, Vidyasirimedhi Institute of Science and Technology, Rayong 21210, Thailand*

[*]*E-mail:* e.vlieg@science.ru.nl, adrian.flood@vistec.ac.th

1. HPLC analysis of solid samples from Viedma ripening deracemization

Samples of the solids were collected over time during the deracemization experiments. For sampling, 40 µL of slurry was taken over time and filtered through vacuum filtration apparatus. The residue was washed with acetone (to remove salicylaldehyde) and dried. Thereafter, this solid was dissolved in 2-propanol and analyzed by HPLC.

HPLC analysis was performed using a chiral AD column via isocratic elution. 20% 2-propanol in heptane containing 0.1% trifluoroacetic acid was used as mobile phase with flow rate of 0.5 mL/min, temperature at 35°C and UV detection at 215 nm. The retention times of *D*-PGA, *L*-PGA, *D*-NAT and *L*-NAT are 9.6, 10.8, 17.9 and 20.0 min, respectively.

2. Additional experimental results

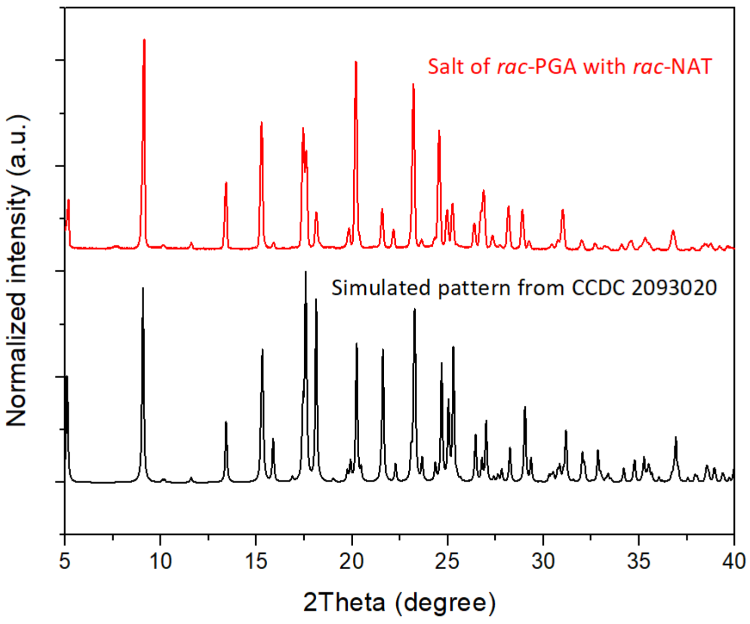

Figure S1. XRPD of simulated pattern from CCDC 2093020 (black) and the pattern of the salt of *rac*-PGA and *rac*-NAT obtained from the experiment (red).

Table S1. Hydrogen bonding information of two polymorphic forms (Form I and II) of the homochiral (*DD*- or *LL*-) salt.

- **Form I**

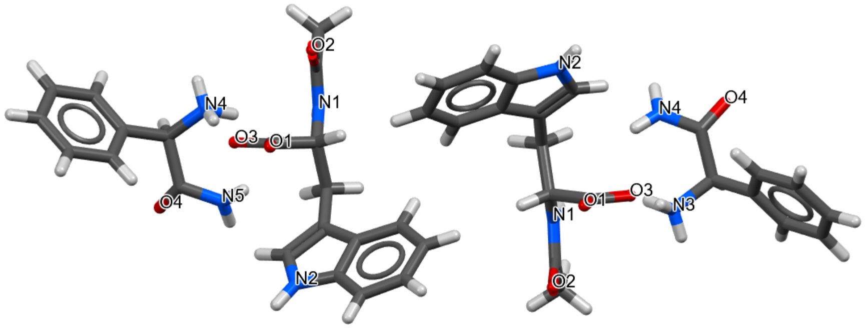

Donor	Acceptor	D-H (Å)	H...A (Å)	D...A (Å)	D-H...A (°)	Type
N1-H1	O1	0.85	2.034	2.830	155.1	intermolecular
N3-H107	O1	0.95	1.754	2.702	173.8	intermolecular
N3-H109	O3	0.88	1.874	2.750	176.2	intermolecular
N4-H106	O2	0.84	1.940	2.782	175.5	intermolecular
N2-H5	O4	0.88	2.173	2.839	132.0	intermolecular
N4-H123	O4	0.88	2.291	3.007	138.5	intermolecular

- **Form II**

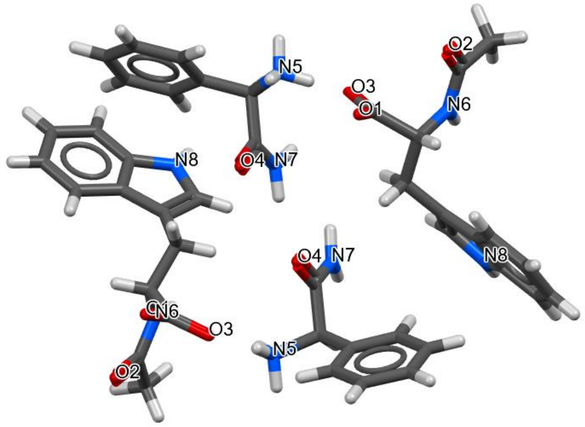

Donor	Acceptor	D-H (Å)	H...A (Å)	D...A (Å)	D-H...A (°)	Type
N6-H6	O1	0.85	2.135	2.894	148.6	intermolecular
N5-H5B	O1	0.89	1.774	2.634	162.6	intermolecular
N5-H5B	O3	0.89	2.701	3.322	128.2	intermolecular
N7-H7B	O3	0.90	2.394	3.087	134.1	intermolecular
N5-H5C	O3	0.86	2.010	2.859	168.5	intermolecular
N5-H5A	O2	0.92	1.925	2.847	175.8	intermolecular
N7-H7A	O4	0.89	1.992	2.867	167.6	intermolecular

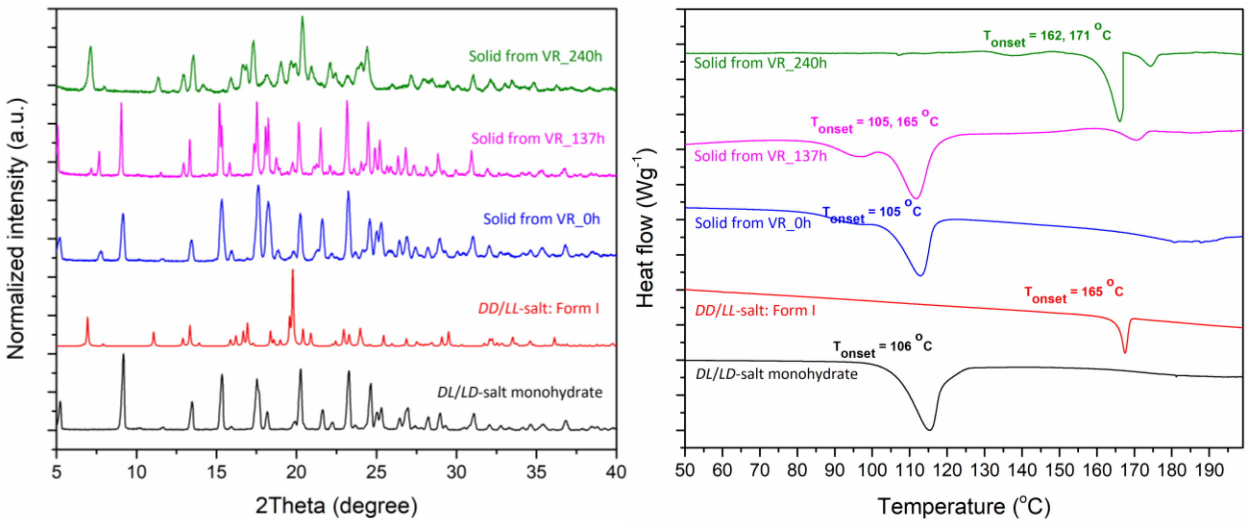

Figure S2. XRPD patterns (left) and DSC thermograms (right) of solid phase obtained from Viedma ripening experiment at 0h (blue), 137h (pink), and 240h (green) compared to the heterochiral (*DL*- or *LD*-) monohydrate salt (black) and a polymorphic form of the homochiral (*DD*- or *LL*-) salt (red) prepared from enantiopure starting materials, which we refer to as form I.

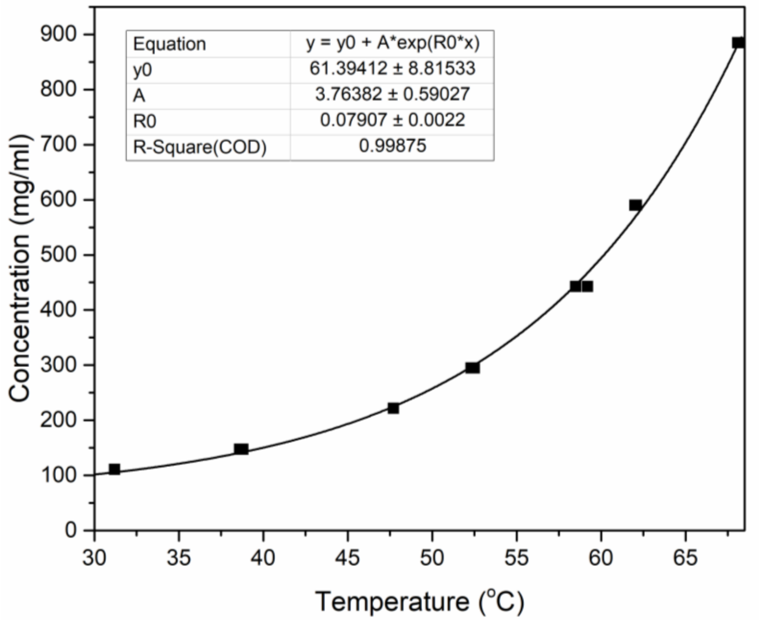

Figure S3. Solubility curve of the heterochiral (*DL*- or *LD*-) monohydrate salt with additional 50% excess of *rac*-NAT in ethanol with the presence of 0.26 mol of salicylaldehyde per 1 mol of PGA species.

Table S2. *ee* of PGA and NAT species of the first and final samples observed from the Viedma ripening experiments using 50% excess of *rac*-NAT. EXP# 2 has a lower suspension density and therefore proceeds faster.

Experiment #	Time (hours)	*ee* of PGA (%)	*ee* of NAT (%)
EXP#1	0	39	-28
	408	-24	-22
EXP# 2	0	22	-17
	136	-7	-13
EXP# 3	0	19	-14
	426	-18	-10

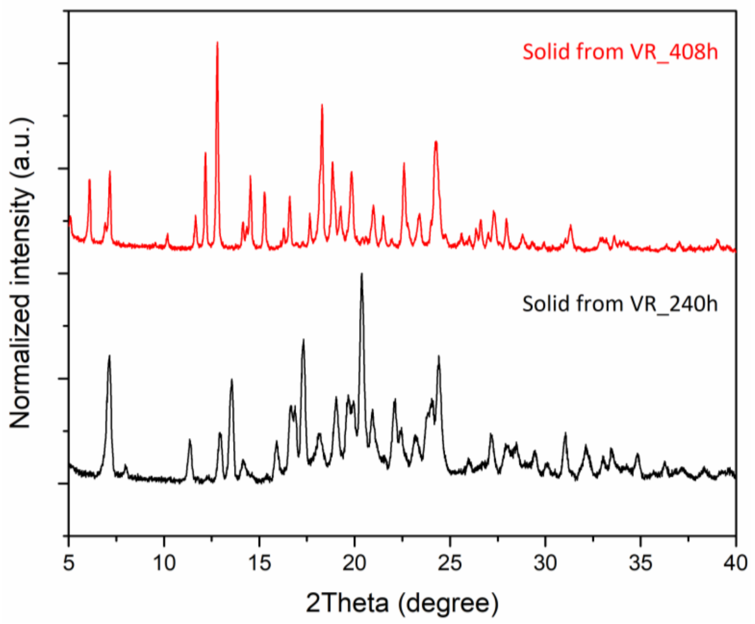

Figure S4. XRPD patterns of solid phase obtained from Viedma ripening experiment at 240h (black), and 408h (red). The additional peaks in the red pattern are registered to the phase of racemic compound.

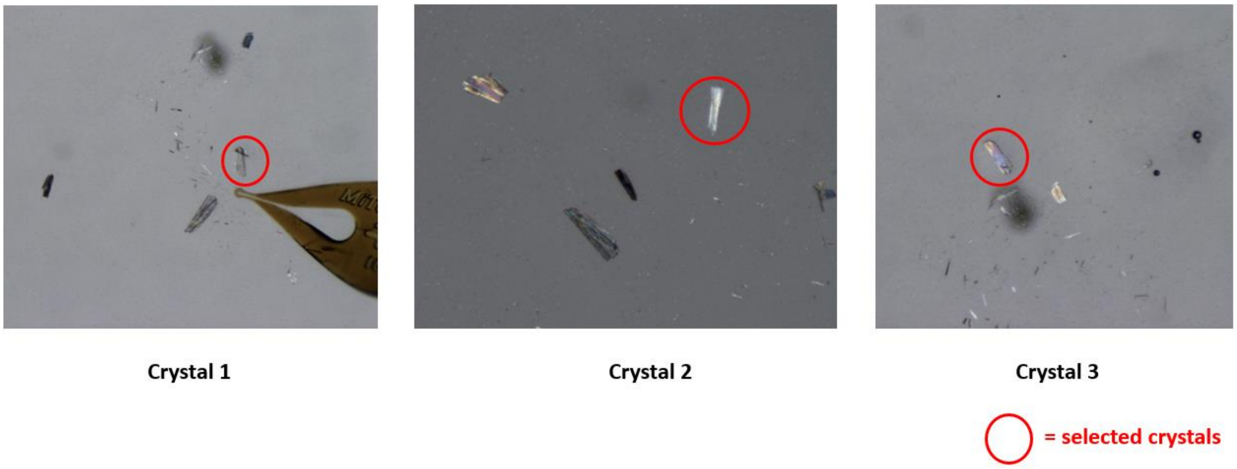

Figure S5. Optical microscope images of the selected crystals of the most stable phase of the salt for HPLC analysis.

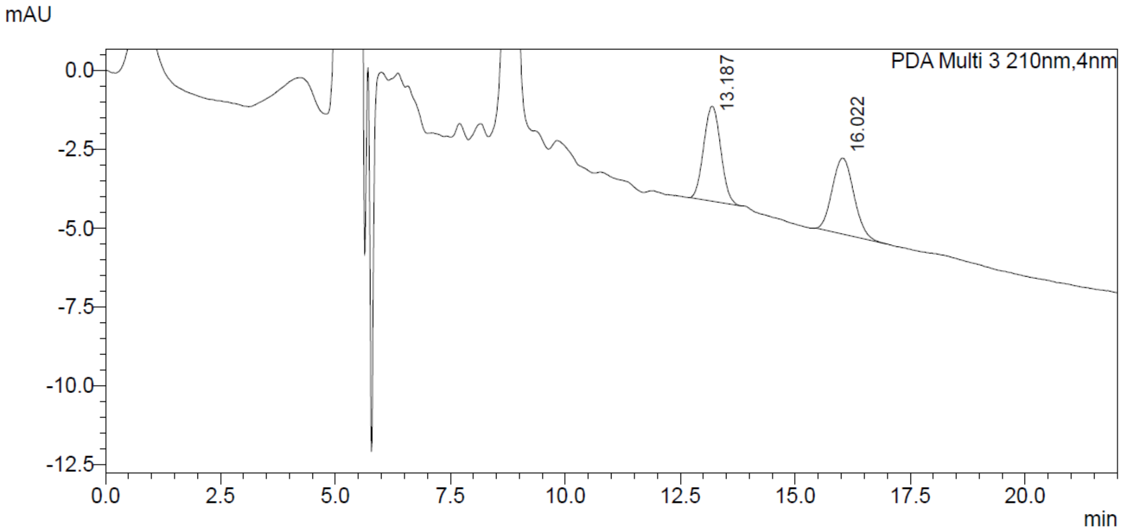

Figure S6. Example of HPLC chromatogram of the selected crystals (the displayed result is 1%*ee* NAT).

Table S3. *ee* of NAT species observed in each crystal of the most stable phase of the salt.

Crystal	*ee* of NAT (%)
1st crystal	1
2nd crystal	2
3rd crystal	0

Note that *ee* of PGA species in the salt cannot be determined using a Lux® 5 μm Amylose-1 column.
